# A Unique Radiation Scheme for the Treatment of High-Grade Non-Metastatic Soft Tissue Sarcoma: The Detroit Medical Center Experience

**DOI:** 10.1080/13577140500397146

**Published:** 2005

**Authors:** James Fontanesi, Michael P. Mott, Michael J. Kraut, David R. Lucas, Peter R. Miller

**Affiliations:** 1 Department of Radiation Oncology University of Mississippi Medical Center 2500 North State Street Jackson MS 39216 USA; 2 Department of Orthopedic Surgery Wayne State University School of Medicine Pathology and Diagnostic Imaging Detroit MI 48201 USA; 3 Department of Medical Oncology Providence Hospital Barbara Ann Karmanos Cancer Institute Detroit MI 48201 USA; 4 Department of Pathology Wayne State University School of Medicine Detroit Medical Center Wayne State University, 4100 John R Detroit MI 48201 USA; 5 Department of Radiology Wayne State University School of Medicine Detroit Medical Center Wayne State University, 4100 John R Detroit MI 48201 USA

## Abstract

*Purpose:*This is the initial report on the utilization of combined photon irradiation followed by a neutron boost irradiation for the initial management of patients with high-grade non-metastatic soft tissue sarcoma (STS). We present data on local control, complications, disease-free survival and overall survival in patients at high risk for local relapse.

*Methods and materials:* Between 1/1/1995 and 10/31/02, twenty-three patients with high-grade non-metastatic soft tissue sarcoma were referred to the Department of Radiation Oncology at the Detroit Medical Center. These patients were referred for consultation due to surgical margin status (tumor within 3mm of surgical margin (*n*=11)), or gross residual disease (*n*=12). There were 14 males and nine females whose ages ranged from 12 to 75 at the time of diagnosis (med=44 years). The most common histology was malignant fibrous histiocytoma (*n*=6), followed by liposarcoma (*n*=5), synovial sarcoma (*n*=4), and angiosarcoma (*n*=2). Twenty-one of 23 patients also received multi-agent multi-cyclic cyto-reductive therapy. Treatment consisted of initial daily photon irradiation delivered either using twice daily fractions of 120 cGy (*n*=10) or once daily 200 cGy/fx (*n*=13).Total photon dose was 36–39.6 Gy. Neutron irradiation was initiated immediately following the photon irradiation and consisted of fraction sizes of 1.0–1.25NGy to a total dose of 6–10 NGy. The neutrons were given once daily. Follow-up is calculated from the day of last radiation treatment.

*Results:* No patient has been lost to follow-up, which has ranged from 18 to 82 months (med=36 months). To date there have been two local relapses and three patients with distant disease development without local relapse. Each of the patients with distant disease has died. The local failures occurred at 9 and 12 months. The 36-month local control is 91%. Thirtysix month disease-free survival was 78%. Overall survival at 36 months was 87%. Three patients had unusual complications consisting of delayed wound healing, and in one of these patients a fracture of the tibia has been noted.

*Conclusion:* The use of this unique radiation sequence post-surgically in patients at high risk for local relapse has resulted in an exciting 36-month local control rate of 91%. The 3-year disease-free survival of 78% and overall survival rate of 87% are exciting but need to mature. The low complication rate is similar to that reported in other large institutional series that have not utilized neutrons. We continue to evaluate the role of combined photon and once-off neutron irradiation in the treatment of patients with high-grade STS that are risk for local recurrence.


*Sarcoma*, September/December 2005; 9(3/4): 141–145

Taylor & Francis

Taylor & Francis Group

**ORIGINAL ARTICLE**

### A unique radiation scheme for the treatment of high-grade non-metastatic soft tissue sarcoma: The Detroit Medical Center experience

JAMES FONTANESI[1], MICHAEL P. MOTT[2], MICHAEL J. KRAUT[3], DAVID R. LUCAS[4], & PETER R. MILLER[5]

[1]*Department of Radiation Oncology, University of Mississippi Medical Center, Jackson, MS 39216,*
[2]*Department of Orthopedic Surgery, Wayne State University School of Medicine, Pathology and Diagnostic Imaging,*
[3]*Department of Medical Oncology, Providence Hospital, Barbara Ann Karmanos Cancer Institute,*
[4]*Department of Pathology, Wayne State University School of Medicine, and* [5]*Department of Radiology, Wayne State University School of Medicine, Detroit Medical Center, Wayne State University, 4100 John R, Detroit, MI 48201, USA*

**Abstract**

*Purpose:* This is the initial report on the utilization of combined photon irradiation followed by a neutron boost irradiation for the initial management of patients with high-grade non-metastatic soft tissue sarcoma (STS). We present data on local control, complications, disease-free survival and overall survival in patients at high risk for local relapse.

*Methods and materials:* Between 1/1/1995 and 10/31/02, twenty-three patients with high-grade non-metastatic soft tissue sarcoma were referred to the Department of Radiation Oncology at the Detroit Medical Center. These patients were referred for consultation due to surgical margin status (tumor within 3 mm of surgical margin ($n = 11$)), or gross residual disease ($n = 12$). There were 14 males and nine females whose ages ranged from 12 to 75 at the time of diagnosis (med = 44 years). The most common histology was malignant fibrous histiocytoma ($n = 6$), followed by liposarcoma ($n = 5$), synovial sarcoma ($n = 4$), and angiosarcoma ($n = 2$). Twenty-one of 23 patients also received multi-agent multi-cyclic cyto-reductive therapy. Treatment consisted of initial daily photon irradiation delivered either using twice daily fractions of 120 cGy ($n = 10$) or once daily 200 cGy/fx ($n = 13$).Total photon dose was 36–39.6 Gy. Neutron irradiation was initiated immediately following the photon irradiation and consisted of fraction sizes of 1.0–1.25 NGy to a total dose of 6–10 NGy. The neutrons were given once daily. Follow-up is calculated from the day of last radiation treatment.

*Results:* No patient has been lost to follow-up, which has ranged from 18 to 82 months (med = 36 months). To date there have been two local relapses and three patients with distant disease development without local relapse. Each of the patients with distant disease has died. The local failures occurred at 9 and 12 months. The 36-month local control is 91%. Thirty-six month disease-free survival was 78%. Overall survival at 36 months was 87%. Three patients had unusual complications consisting of delayed wound healing, and in one of these patients a fracture of the tibia has been noted.

*Conclusion:* The use of this unique radiation sequence post-surgically in patients at high risk for local relapse has resulted in an exciting 36-month local control rate of 91%. The 3-year disease-free survival of 78% and overall survival rate of 87% are exciting but need to mature. The low complication rate is similar to that reported in other large institutional series that have not utilized neutrons. We continue to evaluate the role of combined photon and once-off neutron irradiation in the treatment of patients with high-grade STS that are risk for local recurrence.

### Introduction

The use of neutron radiotherapy in the treatment of STS has been reported by several groups worldwide with varied results [1–3]. Local control rates have been reported from 18 to 90% and may have varied due to treatment intent (initial management or at the time of relapse), surgical margin status (gross residual, microscopically positive residual or close surgical margins), histopathology (high versus low grade), and neutron beam characteristics (deeply penetrating or superficial) [4].

Correspondence: James Fontanesi, M.D., Chairman, Department of Radiation Oncology, University of Mississippi Medical Center, 2500 North State Street, Jackson, MS 39216, USA. Tel: 1 601 984 2550. Fax: 1 601 815 3204. E-mail: jfontane@ci.umsmed.edu

ISSN 1357–714X print/ISSN 1369–1643 online/05/(03-04)0141-5 © 2005 Taylor & Francis
DOI: 10.1080/13577140500397146

While there may be controversy about these factors, certain parameters are accepted as to why neutron beam radiotherapy may have a potential benefit in the treatment of STS. These include the lack of cell cycle positional effect for neutron killing, difference in the oxygen enhancement ratios which favors neutrons in hypoxic environments, and an improved RBE [5]. However, these benefits to cell kill are non-discriminating and are often enhanced in hydrogen-rich tissues, often the same tissues that are responsible for the origin of the STS, and can lead to significant complications. These complications have also been reported to be enhanced with increasing treatment volume and daily neutron fraction size [6].

Although traditional surgical series have usually reported the highest local control, overall and disease-free survivals are not significantly different from modern limb salvage series, with the major cause of death development of distant disease sites [7,8]. However, in reported series, patients treated with either microscopic or gross residual disease had a demonstrable reduction in local control. Recently there has been renewed excitement regarding novel systemic agents that may improve disease-free and overall survival due to new mechanisms of action when compared to more traditional chemotherapy regimens [9,10].

The Detroit Medical Center has had neutron beam capacity since 1984.The characteristics of the neutron beam have been previously described [11]. Beginning in 1995 we instituted a policy to treat selected patients (not eligible/willing to participate in national protocols, not eligible for brachytherapy, having pathological high-grade non-metastatic STS with high risk for local failure based on post-surgical margin status) with a combined photon/neutron radiotherapy treatment course. We undertook this action due to what was felt to be less than desirable local failure rates seen in these patients, in our institution. These patients were not considered for re-operation since the surgery would have resulted in dysfunctional limbs or would require amputation. With an estimated RBE of 4.5, we were able to achieve a combined dose approximately equivalent to 70–80 Gy in 6 weeks. In addition, our initial experience with this combination in the pre-operative setting was very encouraging, in that we were pathologically able to obtain 90–95% tumor necrosis in the resected specimens.

We report on projected 36-month local progression-free, disease-free and overall survival, complications and also report on unusual post-radiation imaging findings believed to be related to the neutron boost radiotherapy.

### Methods and materials

Between 1/1/1995 and 10/31/2002, 23 patients who presented with non-metastatic high-grade soft tissue sarcoma and were not participating in IRB approved clinical trials nor were eligible for radiation therapy that would have included brachytherapy, were referred to the Department of Radiation Oncology at the Detroit Medical Center for evaluation for therapy that included neutron radiotherapy. All patients signed informed consents.

There were 14 males and nine females referred for initial radiotherapy management. Their ages ranged from 12 to 75 years at the time of diagnosis (med = 44 years). Each had undergone surgical extirpation in an effort to obtain clean surgical margins. However, 12 patients were referred with gross residual disease and 11 were referred with microscopically positive/close margins, which were within 3 mm of the rejected margin (Table I).

The most common histology was malignant fibrous histiocytoma (MFH). This was followed by liposarcoma (LP), synovial sarcoma (SS) and angiosarcoma (AS). All pathology was reviewed by one of the authors (DRL) and found to be high grade. Each patient had standard metastatic work-up pre-operatively and found to be without evidence of distant disease. Twenty-one out of 23 received neoajsuvant chemotherapy, which most often contained either an adraimycin- or a gemcitabine-containing regimen.

Following surgical resection, radiotherapy was initiated within 2 weeks until 1999 at which time, due to three wound healing delays, we elected to initiate treatments 4 weeks after surgery. Initial irradiation consisted of either twice daily ($n = 10$) or once daily ($n = 13$) photon irradiation to the tumor bed plus a 5-cm margin. Each BID fraction delivered 1.2 Gy and had an interval of 4–6 h between fractions. Those treated once daily received 200 cGy/fraction. The total dose delivered via the photons ranged from 36 to 39.6 Gy. Neutron therapy was initiated immediately following the photon irradiation. Each fraction was given once daily and delivered 1.0–1.25 NGy. Total neutrons delivered ranged from 6 to 10 NGy (Table II). The neutron treatment field was the tumor bed plus 2 cm. Custom shielding was used where appropriate for both the photon and neutron treatments. This is especially relevant to the unique beam-shaping capacity that is unique to our cyclotron.

Follow-up has been maintained on all patients and is calculated from the last day of radiation treatment. It has ranged from 18 to 82 months

Table I. Patient characteristics.

| | |
|---|---|
| Male | 14 |
| Female | 9 |
| Age at diagnosis | 12–75 years (median = 44 years) |
| Received multi-agent chemotherapy | 21/23 |
| Follow-up | 18–82 months (median = 36 months) |

Table II. Tumor and treatment characteristics.

| | |
|---|---|
| Histology | |
| Malignant fibrous histiocytoma (MFH) | 6 |
| Liposarcoma (LP) | 5 |
| Synovial sarcoma (SS) | 4 |
| All others | 8 |
| Surgical margin | |
| Within 3 mm | 11 |
| Grossly positive | 12 |
| External photons treatment schedule | |
| BID | 10 |
| QD | 13 |
| External photon total dose | 36–39.6 Gy |
| Neutron dose/fraction | 1.0–1.25 NGy |
| Total neutron dose | 6–10 NGy |

(med = 36 months). At routine intervals, physical examinations and radiographic imaging with chest X-rays and CT/MRI imaging of the primary site were carried out. These were reviewed at a multidisciplinary conference held weekly.

Local relapse-free survival and overall survival were calculated from the last day of neutron therapy. Local relapse was described as any pathological finding of recurrent disease in the field of photon irradiation. The 36-month local control, disease-free and overall survival were calculated using the Kaplan–Meier method [13].

### Results

### *Local control*

At last follow-up there has been two local failures. The first patient, who failed at 9 months, was treated with microscopically positive surgical margins. The failure occurred at the edge of the photon field. The second patient, who failed at 12 months, also treated with microscopic disease, failed within the neutron field. The local progression-free survival at 36 months is 91%.

### *Disease-free survival*

Three patients, two with microscopic disease and one with gross residual disease, have developed distant failure while maintaining local control. All distant disease was diagnosed within 18 months of completion of the irradiation. These patients have died of their disease. The 36-month disease-free survival is 78%.

### *Overall survival*

No patient has been lost to follow-up. At the time of this report the three patients with distant failures have died. The 36-month overall survival is 87%.

### *Complications*

There have been three patients who have suffered from unusual delayed wound healing. These patients prompted our decision to delay initiation of irradiation from 2 to 4 weeks post-operatively. Two of these patients also had received gemcitabine as a radio-sensitizer during their photon irradiation. In addition, one of the two receiving gemcitabine also was noted to have a radiographic finding of a tibeal fracture.

All other patients have exhibited RTOG grade I/II skin toxicity. Four patients have also reported to have reduced range of motion.

### *Radiographic follow-up*

Early in our experience we noted patients, in whom MRIs were obtained in routine post-therapy follow-up, who had noticeable T2 signal changes often lasting 6 months post-treatment. In the first three patients in which this was noted biopsies were obtained and reviewed by one of the authors (DRL). In each case no tumor was found. There were changes consistent with soft tissue injury with significant cellular damage. After these initial patients, no further biopsy evidence was attempted unless there was clear evidence of new growth, not just changes consistent with edema and soft tissue injury.

### Discussion

Local control is but one of the issues paramount in the treatment of non-metastatic high-grade soft tissue sarcoma. If in fact local control was the overriding factor in survival, we would, to this day, be using radical amputation as the standard of care. However, it was reported that the high degree of local control that these radical procedures established did not translate into improved disease-free survival [14,15]. This problem led to a search for ways to reduce this problem of distant disease development, but also to seek improved functional outcomes rather than resort to amputative surgical interventions. Pioneering work from several groups demonstrated that 'limb salvage' therapy could be accomplished with the combined modality therapy that is the hallmark of modern care for STS [7,8].

Institutional series soon began to report local control rates of 65–80% and without a loss in overall survival when compared to the amputation series. Herein lies the next great hurdle: how to approximate the amputation series local control rates while maintaining or improving on the recently reported combined modality series. The systemic approaches being investigated include the use of newer novel systemic agent such as anti-angiogenesis and other unique acting agents/drugs [9,10].

Early on the unique nature of STS therapy caught the attention of the neutron radiotherapy community. STS are a collection of various tumors of various cellular origins, often capable of rapid growth and often with significant hypoxic populations. Thus STS became an obvious tumor system in which the unique properties that neutrons possess were thought to perhaps give an advantage over photon irradiation.

Our reported series differs significantly from the only other ongoing neutron-based experience in the USA [1]. The University of Washington, Seattle, recently updated their series of STS patients who have received neutrons as their radiotherapy management. They reported a projected 4-year local relapse-free survival of 68%. Of interest is the fact that 19 of the reported patients had photon doses similar that those reported in our series. Unfortunately these patients are not broken out from the entire group for analysis to determine if the photons conferred any local control advantage. The major difference in the two series is the total neutron doses delivered. In the Washington series the median neutron dose was 18.3 NGy, which is almost double that which we delivered. Our concept of neutron radiation is more in line with the European neutron experience in which the neutrons are used more as a 'boost' to supplement the photon irradiation. These European centers have reported widely varying local control rates of 18–94% [16–18].

The use of neutrons, with its higher RBE, lack of concern for the cell cycle position of target cells, and disregard for the hypoxic status, while potentially more lethal for STS cells is also capable of significant normal tissue damage especially those which are hydrogen rich. Our complication rate (RTOG Gr. III/IV) of 13% is similar to that reported in most series regardless of type of radiation used, whether it includes external beam alone, brachytherapy or neutrons. The University of Washington reported that 21% of patients had serious chronic radiation-related complications. The European series reported significant complications in the range of 7–59%. However, many of the lower rates for complications came from European centers in which the neutrons were used as a boost to supplement photon irradiation [6,17,19]. Although our center and others have reported on the increased risk of complications based on increased neutron delivered per fraction, the impact of aggressive surgical intervention and chemotherapy prior to and after irradiation is very difficult to asses [6] and may also play a significant role in complication development.

Several series have also reported that the treatment volume may play a role in increased complications [1,2]. We agree that this may play an important role in complication development and is one of the main reasons why it was elected to use the neutrons in a 'boost' environment, as a way to deliver treatment to the site of the tumor bed with a limited margin. What was also unique to this series was the lack of influence that the surgical margin had on local control. This, in addition to the lack of influence that histological subgroup had on local control, differs from the recently reported series by Zagars and Ballo [20]. We, however, agree that dose equivalents above 60 Gy are needed for patients with 'dirty' margins or gross residual disease. This is in keeping with several other reports of series that have suggested improved local control with increased dose [21,22]. There was also our theoretical advantage of overall time reduction to 5.5–6 weeks to complete therapy in which, using an RBE of 4.0, we were able to deliver an equivalent dose of up to 80 Gy, with no apparent increase in complications.

The remaining focus of this report was to describe the unusual and long-lasting signal abnormalities that were seen in these patients on post-therapy routine surveillance imaging of the primary area. The finding of persistent soft tissue edema has previously been reported for hyperfractionated photon irradiation and for neutron irradiation; however, as this is the first report that we can identify of the combination of theses two modalities in the treatment of STS, it is not surprising that it occurred [23]. Initial concern led to a biopsy in the first three patients in whom this finding was apparent, all with pathological confirmation of soft tissue injury without local tumor relapse.

### Conclusion

We report on a series of 23 patients treated at the time of initial presentation with a unique radiation combination. Although photon irradiation followed by daily neutron irradiation has been reported from our institution in the management of prostate cancer, this is the first report that we can identify in which hyperfractionated irradiation has been used in combination with neutrons in the treatment of STS. This select group of patients, all with high-grade STS and at high risk from local relapse, have a projected local control rate at 36 months of 91%. While encouraging, it will need further maturation. The 3-year estimated disease-free and overall survival of 78 and 87%, respectively, are also encouraging but with the same concern regarding length of follow-up.

The grade III/IV complication rate of 13% is also acceptable but we have not had significant complication, especially related to wound healing, since we decided to delay initiation of irradiation post-operatively from 2 to 4 weeks. We have also documented long-term unusual imaging findings of soft tissue injury most likely due to soft tissue edema. These findings have been associated with the treatment fields, especially the neutron portals.

We continue to use this treatment regimen for patients not eligible or refusing entry into national protocols, and those not eligible for brachytherapy. It has resulted in an exciting early local control rate and promising relapse-free and overall 3-year survival, although this enthusiasm must be tempered with the knowledge that these data will need further maturation. However, based on this report and others from various neutron facilities worldwide, we believe that a randomized trial to evaluate its efficacy is warranted.

### References

1. Schwartz DL, Einick J, Bellon J, Laramore GE. Fast neuron radiotherapy for soft tissue and cartilaginous sarcomas at high risk for local occurrence. Int J Radiat Oncol Biol Phys 2001;50(2):449–456.
2. Schonekaes KG, Prott FJ, Micke O, Willich N, Wagner W. Radiotherapy on adult patients with soft tissue sarcoma with fast neutrons or photons. Anticancer Res 1999;19(3B): 2355–2359.
3. Steingraber M, Lessel A, Jahn U. Fast neuron therapy in treatment of soft tissue sarcoma – the Berlin-Buch study. Bull Cancer Radiother 1996;83(Suppl):122–124s.
4. Schwartz R, Krull A, Steingraber M, et al. Neurotherapy in soft tissue sarcomas: A review of European results. Bull Cancer Radiotherapy 1996;83(Suppl):110–114s.
5. Wagner W, Bottcher HD, Gohde W, Harle A. Radiosensitivity of osteosarcomas on nude mice. A comparative study of treatment with fast neutrons and cobalt 60. Strahlenther Onkol 1989;165(7):515–516.
6. Duncan W, Arnott SJ, Jack WJ. The Edinburgh experience of treating sarcomas of soft tissues and bone with neutron irradiation. Clin Radiol 1986;37(4):317–320.
7. Chao C, McMasters KM, Edwards MJ. Advances in the treatment of soft-tissue sarcomas. J Ky Med Assoc 2002;1:10–16.
8. Lejeune FJ, Pujol N, Lienard D, et al. Limb salvage by neoadjacent isolated perfusion with TNFalpha and melphalan for non-resectable soft tissue sarcoma of the extremities. Eur J Surg Oncol 2000;26(7):669–678.
9. Beech D, Pollock RE, Tsan R, Radinsky R. Epidermal growth factor receptor and insulin-like growth factor-I receptor expression and function in human soft tissue sarcoma cells. Int J Oncol 1998;12(2):329–336.
10. Fishman D, Galitzki L, Priel E, Segal S. Epidermal growth factor regulates protein kinase A activity in murine fibrosarcoma cells: Differences between metastatic and nonmetastatic tumor cell variants. Cancer Res 1997;57(23): 5410–5415.
11. Maughan RL, Yudelev M. Physical characteristics of a clinical d(48.5)+Be neutron therapy beam produced by a superconducting cyclotron. Med Pys 1995;22(14):59–65.
12. Hoperwel JW, Barnes DW, Robbins ME, Sansom JM, Knowles JF, Van de Aardweg G.J. The relative biological effectiveness of fractionated dosed of fast neutrons (42 MeVd–Be) for normal tissues in the pig. I. Effects on the epidermis and dermal vascular/connective tissues. Br J Radiol 1998;61:928–938.
13. Kaplan EL, Meier P. Nonparametric estimation from incomplete observations. J Am Stat Assoc 1958;53:457–481.
14. Potter DA, Kinsella T, Glatstein E, et al. High-grade soft tissue sarcomas of the extremities. Cancer 1986:58(1): 190–205.
15. Geer RJ, Woodruff J, Casper ES, Brennan MF. Management of small soft tissue sarcoma of the extremity in adults. Arch Surg 1992;127(11):1285–1289.
16. Richard F, Renard L, Wambersie A. Results of neutron therapy of locally advanced soft tissue sarcomas at louvain-la-Neuve. Strahlentherapie 1985;161(12):796–796.
17. Schmitt G, Pape H, Zamboglou N. Long term results of neutron- and neutron-boost iiradiation of soft tissue sarcomas. Strahlenther Onkol 1990;166:61–62.
18. Pickering DG, Stewart JS, Rampling R, Errington RD, Stamp G, Chia Y. Fast neutron therapy for soft tissue sarcoma. Int J Radiat Oncol Biol Phys 1987;13(10): 1489–1495.
19. Hubener KH, Schwartz R, Gleisberg H. Neutron therapy of soft tissue sarcomas and status report from the radiotherapy department of the Hamburg University hospital. Strahlenther Onkol 1989;165:309–310.
20. Zagars GK, Ballo MT. Significance of dose in postoperative radiotherapy for soft tissue sarcoma. Int J Radiat Oncol Biol Phys 2003;56:473–481.
21. Wolfson AH, Benedetto PW, Mnaymneh W, et al. Does a radiation dose-response relation exist concerning survival of patients who have soft-tissue sarcomas of the extremities? Am J Clin Oncol 1998;21:270–274.
22. Dinges S, Budach V, Budach W, et al. Local recurrences of soft tissue sarcomas in adults: A retrospective analysis of prognostic factors in 102 cases after surgery and radiation therapy. Eur J Cancer 1994;30:1636–1642.
23. Richardson ML, Zink-Brody GC, Patten RM, Koh WJ, Conrad EU. MR characterization of post-irradiation soft tissue edema. Skeletal Radiol 1996;25(6):537–543.

